# A Psychometric Evaluation of the Guilt and Shame Experience Scale (GSES) on a Representative Adolescent Sample: A Low Differentiation between Guilt and Shame

**DOI:** 10.3390/ijerph17238901

**Published:** 2020-11-30

**Authors:** Klara Malinakova, Jana Furstova, Michal Kalman, Radek Trnka

**Affiliations:** 1 Olomouc University Social Health Institute, Palacký University Olomouc, 771 11 Olomouc, Czech Republic; jana.furstova@oushi.upol.cz (J.F.); trnkar@volny.cz (R.T.); 2 Faculty of Physical Culture, Institute of Active Living, Palacký University Olomouc, 771 11 Olomouc, Czech Republic; michal.kalman@upol.cz; 3 Science and Research Department, Prague College of Psychosocial Studies, Hekrova 805, 149 00 Praha 4-Háje, Czech Republic

**Keywords:** GSES, guilt, shame, psychometric evaluation, adolescents, religious attendance

## Abstract

The Guilt and Shame Experience Scale (GSES) is a new, brief self-report instrument for assessing experiences of guilt and shame. It includes two distinct scales: feelings of shame and feelings of guilt. The present report focuses on results from a final validation study using a nationally representative sample of 7899 adolescents (M age = 14.5 ± 1.1 years, 50.7% boys) who participated in the 2014 Health Behavior in School-aged Children study. For factor analysis, the dataset was divided into two groups. One group (*n* = 3950) was used for the Exploratory Factor Analysis (EFA) and the second (*n* = 3949) for the Confirmatory Factor Analysis (CFA). The EFA results in a one-factor model of the GSES scale, while the CFA suggests a two-factor solution mirroring two scales, feelings of shame and feelings of guilt. Both models have a good fit to the data, and the scale also showed high internal consistency (Cronbach’s alpha = 0.89). A nonparametric comparison of different sociodemographic groups showed a higher disposition for experiencing guilt and shame among girls, students of the ninth grade, and religious respondents. A comparison of the results to previously published results obtained from adults indicates that adolescence is a developmental period involving low differentiation between moral emotions like guilt and shame compared with adulthood. Moreover, positive association with religious attendance shows a need of addressing these issues in a pastoral care setting.

## 1. Introduction

Guilt and shame are complex, self-conscious emotions linked to one’s responsibility of meeting social standards and rules [1]. The research of these emotions is exceptionally important, because their experience has been found to be related to various health-related aspects of quality of life. Shame was found to be significantly related to depressive symptoms and cognitions [2] and also interlinked with the occurrence of different forms of abuse, emotional neglect, hostility, problematic alcohol and drug use, eating disorders, nonsuicidal self-harming, and repressed anger in adolescents [3,4,5]. Shame also partially mediated the positive relationship between distorted obsessive–compulsive beliefs and depression [6]. Additionally, shame-proneness impaired empathy, which may result in various interpersonal difficulties [7]. In contrast to shame, guilt seems to be a relatively benign emotion when experienced alone, but becomes maladaptive when being excessive, ruminative, and fused with shame [8,9]. Taken together, the above-outlined negative health outcomes of shame highlight the importance of further research on guilt and shame in the field of public health (see also [10,11]).

To begin with, let us have a closer look at differences in definitions of guilt and shame. Both emotions are experienced when a person violates some moral or social norms, while realizing that this transgression is noticed by other people [8]. However, shame and guilt are distinct emotions with different implications for motivation and adjustment [3]. Guilt is concerned with the negative evaluation of a specific behavior in a particular situation, resulting in a desire to confess, apologize, and repair [12]. In contrast, shame involves the negative evaluation of the self and elicits a desire to vanish or escape [12]. In other words, guilt is a negative feeling much more related to the specific event, rather than to the self. In contrast, shame involves a global negative feeling about the self in response to some misdeed or failure that is often triggered by a social event in which a drop of personal status or feelings of rejection are perceived [3,13].

Some factors have already been associated with a higher disposition for experiencing feelings of guilt and shame in adolescents, for example, gender [14], childhood maltreatment [15], parenting style [16], or bullying [17]. However, we could presume that other factors will also be involved, including religiosity and spirituality. This presumption can be supported by a previous study reporting that religious scrupulosity was negatively associated with one’s well-being [18] and that the higher levels of religious participation in early adolescence could result in a higher moral sensitivity, which can consequently contribute to feelings of guilt regarding everyday transgressions [19].

The growing need for creating a more solid scientific background in this area also stresses the key importance of having valid instruments suitable for this kind of research. At this moment, there are three types of self-report measures of guilt and shame available: scenario-based, adjective-based and statement-based measures. Scenario-based measures ask participants to read hypothetical scenarios and select how they would most likely act, think, and feel from a given set of responses. Examples of scenario-based measures are, the Test of Self-Conscious Affect—3 (TOSCA-3) [20] or the Guilt and Shame Proneness scale (GASP) [21]. Scenario-based measures are relatively time-demanding as they require the detailed reading of a hypothetical scenario by participants. Thus, their use in large population or epidemiological surveys is obviously limited. Furthermore, Cohen and Wolf [21] pointed out that the TOSCA-3 has some limitations, for example, that emotional and behavioral responses to transgressions are confounded in the items’ focuses.

Adjective-based measures, for example, the Personal Feelings Questionnaire-2 [22], are based on participants’ rating of the frequency of experiencing guilt and shame. The participants are asked questions like “Indicate to what extent you feel this emotion” and are provided with particular adjectives representing specific discrete emotions, for example, guilt and shame. Adjective-based measures have been criticized because of the detachment of guilt and shame from the specific contexts in which they occur and with no reference to the specific features of these emotions, that is, phenomenological, motivational, and behavioral features [23].

Statement-based measures ask participants to rate how much they agree with a statement describing the experience of shame and guilt. The Brief Shame and Guilt Questionnaire for Children (BSGQ-C) [24], the Adolescent Shame-Proneness Scale (ASPS) [25], or the State Shame and Guilt Scale (SSGS) [26]—a 15-item self-report scale of “in-the-moment (state) feelings” of shame, guilt, and pride, which is also available in a shorter 8-item version [23]—are examples of statement-based measures. However, these measures have certain disadvantages, like a focus only on shame (ASPS), on the child population (BSGQ-C), or limitations concerning the item structure (SSGS), that is, the use of brief phenomenological descriptions of shame and guilt, where the statements are relatively general (e.g., “I feel small”, “I feel humiliated, disgraced”) and lack a deeper contextualization of discrete emotional experiences. Therefore, in order to further explore the psychological mechanism of self-conscious emotions and promote a more appropriate assessment of them, an improved measure of guilt and shame is needed.

The Guilt and Shame Experience Scale (GSES) [27] is a recent statement-based measure of guilt and shame consisting of eight items. This questionnaire is based on items describing the experience of guilt and shame in a more detailed and contextualized manner (e.g., “I blame myself for things that other people do not think of”, “I feel the need to explain or apologize for the reasons for my actions”, or “I’m losing hope that I will ever be a good person”). The GSES was validated on the adult Czech population with very good psychometric results [27] and could thus represent a useful and easy-to-use measure of guilt and shame. However, so far, no validation has been done on the adolescent population. Therefore, the main aim of the present study was to assess the psychometric characteristics of the GSES and to obtain reference norms in a representative sample of Czech adolescents. Moreover, given the above-mentioned research gap in associations of guilt and shame with adolescent religiosity, religious attendance and importance of faith were also considered in the present study.

## 2. Materials and Methods

### 2.1. Participants and Procedure

A nationally representative sample of Czech adolescents was gathered through the 2018 Health Behavior in School-aged Children (HBSC) study. This international study examines health and health-related behavior and socioeconomic determinants in children in 48 European and North American countries. In order to achieve representativity of the sample and in line with the HBSC study protocol, schools were first stratified by region, school size, and type of school (primary schools vs. secondary schools). Consequently, 227 schools were randomly selected from each quota and contacted. Of these schools, 7 refused to participate (i.e., the school response rate was 97%) and were substituted by nearby schools of similar size in their close neighborhood. In the next step, classes from the 5th, 7th, and 9th grades, which are in general corresponding to the age categories of 11-, 13- and 15-year-olds, were randomly selected, one class from each grade per school. Thus, between May and June 2018, we obtained data from 13,885 pupils (response rate 86.4%). The most frequent reasons for nonresponse were illness (8.87%) or other reasons for absence (4.18%), and only 84 children (0.5%) refused to participate in the survey. Of this sample, 238 questionnaires were excluded because of the problems with internal consistency and due to missing responses to key questions. Above that, another 270 respondents were excluded because their age lay outside the age range of the specific class. Thus, the sample involved 13,377 respondents.

Data were gathered through online surveys. Instructions were given by trained administrators with no teachers present in the classroom in order to reduce response bias. Respondents had one school lesson (45 minutes) to complete the questionnaire. The GSES questionnaire was administered only to adolescents from the 7th and 9th grades, that is, to 8997 respondents. Furthermore, questionnaires lacking an answer on any of the GSES items were excluded. This led to a final sample of 7899 respondents (mean age = 14.5, SD = 1.1, 50.7% boys).

Participation in the survey was anonymous and voluntary. The Czech legislative system does not require written informed consent for participation in questionnaire surveys. Thus, we obtained the consent from the school management of all participating schools. Consequently, the school management informed the parents of the pupils in advance, and they could decide if their child would not participate in the survey. Moreover, participation in anonymous questionnaire surveys is covered by a so-called “general consent” that the Czech schools collect from the pupils’ parents/legal guardians at the beginning of each school year. The study design was approved by the Institutional Research Ethics Committee of the Faculty of Physical Culture, Palacký University Olomouc, with the reference No. 9/2016. The survey was conducted under the auspices of the Ministry of Education, Youth and Sports of the Czech Republic and the World Health Organization Country Office in the Czech Republic.

### 2.2. Measures

The original GSES was presented by Malinakova et al. [27]. This research tool was developed following the review of relevant literature and consultations with professionals in the area of psychology and psychotherapy. In the original form, it contained 10 items. However, a psychometric evaluation revealed better characteristics for an 8-item version, which was used in this research. It is a self-assessment measure of one’s experiences of guilt and shame. The eight items are divided into a guilt subscale and a shame subscale, each containing four items. Following the introductory question: “To what degree do you agree with the following statement”, for each item, the respondents answer questions using a four-point scale ranging from “not at all” (1) to “significantly” (4). The overall score from the GSES is computed by summing the responses to all items, so it ranges from 8 to 32, with a higher score corresponding to higher experiences of guilt and shame. The order of items was adjusted according to recommendations of the original article [27]. The whole questionnaire is attached herein as Supplementary File S1—the GSES scale.

Importance of faith was assessed by a single question: “How important is faith for your life?”, which was introduced from Slovak HBSC studies. Possible answers ranged from “not at all” (1) to “absolutely” (7). For the purpose of further analysis, the variable was dichotomized, so respondents who scored higher than 4 points (“Neither important, nor unimportant”) were considered to be those for whom faith is important.

Religious attendance was also assessed with a single question that has been used in the last two Czech HBSC studies: “How often do you go to church or to religious sessions?”. Possible answers ranged from “not at all” (1) to “several times a week” (6). Respondents who reported that they attend religious sessions at least once a week (5 or 6) were considered to be attending.

Gender, age, and other basic sociodemographic characteristics were obtained by the questionnaire.

### 2.3. Statistical Analyses

All statistical analyses were performed in the statistical software R 3.6.0 (R Development Core Team, Auckland, New Zealand) and IBM SPSS Statistics, version 21 (IBM, Armonk, NY, USA). The distribution of individual items and the raw score were executed with histograms. Normality of the data was tested with the Shapiro–Wilk’s test. Since the data was not normally distributed, nonparametric methods were used for the statistical analyses. The correlation between the scale items was evaluated with polychoric correlations. The Wilcoxon rank-sum test was used for comparison of two groups (gender and grade differences, importance of faith). Comparison of multiple groups (religious attendance) was done using the Kruskal–Wallis test (i.e., nonparametric ANOVA) with Bonferroni correction.

For the purposes of factor analysis, the dataset (*n* = 7899) was split into two halves using a random permutation of the respondents’ order. Thus, two sets of data with almost the same characteristics were created and further used: the first one (*n* = 3950) was used for Exploratory Factor Analysis (EFA) and the second (*n* = 3949) for Confirmatory Factor Analysis (CFA). Several methods have been used to determine the number of factors: the Kaiser (K1) criterion, scree plot, Parallel Analysis (PA), and the Minimum Average Partial (MAP) test. The PA and MAP were based on a polychoric correlation matrix using the R package random.polychor.pa. The PA was calculated by simulation of 1000 random matrices of permutated data. The polychoric correlations were used in both the EFA and the CFA analyses. In the EFA with oblique rotation (Oblimin), optimization was performed with the weighted least squares method. Parameter estimation in the CFA was executed using the diagonally weighted least squares method. The comparison of the nested CFA models was carried out with the scaled difference chi-square test using the Satorra–Bentler (2001) approximation. For the EFA and the CFA computing, the R Psych and R lavaan packages were used. Cronbach’s alpha and McDonald’s omega coefficients were employed to assess the internal consistency of the scale.

## 3. Results

### 3.1. Comparison of GSES Scores in Different Groups of Adolescents

The comparison of the mean scores of the whole GSES scale and its two subscales—shame and guilt—between different groups of adolescents is presented in Table 1. Differences in the mean scores within gender groups and grades were assessed with the Wilcoxon rank-sum test, while the importance of faith in God and religious attendance were assessed with the Kruskal–Wallis test (nonparametric ANOVA).

The results shown in Table 1 indicate significant differences in the values of guilt and shame between the various groups of adolescents. Girls reached significantly higher levels of both guilt and shame than boys (*p* < 0.001, with the effect size of Cohen’s d and eta-squared being d = 0. 47–0.54 and e = 0.05–0.07, respectively). There was no significant difference found in shame between the seventh and the ninth grades of school (age groups of 11 and 13 years old); the mean value of guilt was found to be slightly higher in the ninth grade (*p* = 0.002, with low effect size of d = 0.07 and e = 0.001). Religious belief was associated with both subscales, guilt and shame. Those adolescents who reported that faith in God was not important at all scored significantly lower in guilt and shame than all other adolescents (*p* < 0.001, effect size: d = 0.16–0.18, e = 0.006–0.008). Analogously, those adolescents who never go to church had significantly lower values of guilt and shame than all other adolescents (*p* < 0.001, effect size: d = 0.27–0.29, e = 0.019–0.021).

### 3.2. Psychometric Properties

#### 3.2.1. Verification of Factor Structure

A total of three datasets were used for the statistical analyses: the whole dataset (*n* = 7899); and two subsets of the whole dataset obtained by a random permutation of the respondents’ order, which was then split into two halves (the first used for EFA, *n* = 3950, the second used for CFA, *n* = 3949). The descriptive statistics of the three datasets are presented in Table 2 and show that the characteristics of the three data sets are very similar.

The correlation between the individual items of the GSES scale in the whole dataset was medium to high, with values of 0.32–0.65 (see Table 3). Thus, Oblimin rotation was used in the Exploratory Factor Analysis (EFA).

In the first dataset with *n* = 3950, there was only one eigenvalue greater than one; the parallel analysis suggested the extraction of two factors, while the MAP method recommended the extraction of one factor. Therefore, a single-factor model and a two-factor model of the GSES scale were examined. The baseline conditions for using factor analysis [28] were met by the statistically significant result of Bartlett’s test of sphericity (χ²(28) = 14,818.2; *p* < 0.001) and the value of the Kaiser–Meyer–Olkin criterion > 0.8 (KMO = 0.91). The results of EFA on the matrix of polychoric correlations are presented in Table 4. The first two eigenvalues are 4.77 and 0.30, and the first component describes 59.7% of the variability in the data, while the second describes only 3.7% of the variability. Moderate-to-high factor loadings arise for all items in the one-factor model of GSES; all items show a satisfactory communality h2 and a satisfactory correlation with the raw score. In the two-factor model, item 6 incorrectly loaded on the shame factor, and items 1 and 8 had double loadings on both factors. The guilt factor thus consisted of only two items. All items showed a satisfactory correlation with the raw score. According to the EFA results, the single-factor model of the GSES scale performed better.

#### 3.2.2. Confirmatory Factor Analysis

The second half of the permuted data (*n* = 3949) was used for the CFA analyses. Table 2 shows the descriptive characteristics of the dataset. The CFA was based on the matrix of polychoric correlations. Two models were verified (see Table 5)—the original two-factor model presented in Malinakova et al. [27] and the single-factor model acquired in the EFA. In the single-factor model with all eight items, the loadings were moderate to high (with values of 0.70–0.83). The model showed a good fit to our data, with acceptable values for all monitored parameters. In a two-factor model, the shame factor contained items 1, 3, 5, and 7, and the guilt factor comprised items 2, 4, 6, and 8. In this two-factor model, the loadings were slightly higher than in the single-factor model (with values of 0.71–0.84). The model showed a good fit to our data, comparable to the single-factor model. However, the scaled difference chi-square test resulted in *p* < 0.001, which shows the two-factor model explained the data significantly better.

In order to enhance the power of the tests, all of the following analyses were performed on the complete dataset (*n* = 7899).

#### 3.2.3. Reliability

The internal consistency analysis of the whole eight-item GSES questionnaire resulted in a Cronbach’s alpha of 0.89 (95% CI 0.89–0.89). When the scale was divided into two subscales, the Cronbach’s alpha values were 0.80 (95% CI 0.79–0.80) for the shame factor and 0.83 (95% CI 0.83–0.84) for the guilt factor. McDonald’s omega coefficient for the whole scale reached 0.92. These values indicated that the GSES scale acquires a sufficiently high reliability in the population of Czech adolescents.

## 4. Discussion

The aim of this manuscript was to psychometrically assess the characteristics of the GSES scale on a representative sample of Czech adolescents. With regards to the sociodemographic comparison, girls showed a significantly higher disposition for experiencing guilt and shame than boys, and ninth-grade students reported a higher disposition for feelings of guilt than seventh-grade students. Adolescents who reported that faith is not important for them and nonreligious respondents achieved significantly lower scores in both the guilt and shame subscales. With regard to psychometric evaluation, individual items of the GSES scale were mostly moderately correlated. The EFA suggests a single-factor solution, while the CFA prefers a two-factor model. However, both one- and two-factor models showed a good fit with the data. A reliability analysis showed good internal consistency for both the whole scale as well as for the subscales separately. These findings partly correspond to those of a national representative adult Czech sample [27].

Very interestingly, compared to the original study performed on an adult sample, where the two-factor structure fit the data [27], the present study on adolescents found a good fit with the data for both one- and two-factor models. This difference may be explained by a possible low differentiation between the experience of guilt and shame in the period of adolescents. For adolescents, it may be more difficult to distinguish slight differences between feelings following their own misconduct in a particular situation following the effort to repair this misconduct (i.e., guilt) and feelings following a misdeed or unwanted *faux pas* involving desire to vanish or escape (i.e., shame). Adults can be expected to have more personal experiences with guilt and shame due to their higher age. Therefore, adults can be hypothesized to have a better ability to distinguish between guilt and shame. More research is needed in this field, especially longitudinal studies.

Regarding the higher levels of perceived guilt and shame among students of the higher grade, our findings are in line with a number of studies that identified age as an important covariate in guilt and shame analysis. Our findings differ from those of Gambin and Sharp [29], who reported a negative correlation between age and feelings of guilt and shame; however, these authors analyzed substantially smaller samples with a prevalence of women and a broader age range, which could have influenced the results. On the other hand, our findings resonate with research on emotional regulation showing exaggerated responses to emotion in mid-adolescents (age 15 to 18) compared with younger and older individuals [30].

Furthermore, our finding of a higher disposition for experiencing feelings of guilt and shame in girls is in line with the findings reported in the original article describing the psychometric analysis of the GSES on an adult national Czech sample [27], and is also in line with recent findings on adolescent samples, which reported that compared to boys, girls showed a higher disposition for experiencing shame [31], guilt [29], or both of these emotions [14]. A possible explanation could be associated with social roles, as women are usually expected to be more nurturing and empathetic [32]. In the sample of older adults, women were found to show greater levels of shame (specifically behavioral and bodily shame) and lower levels of self-esteem than men [13]. Moreover, the gender differences found could also be associated with the developmental trajectories of adolescent negative emotional experiences, as Maciejewski and van Lier [33] described that girls reported more sadness in general and experienced steeper declines in happiness compared with boys.

Adolescents who in our study reported that faith is important in their life and regularly attended religious services showed higher levels of both guilt and shame. There are only a few studies that have thus far examined this relationship, and their results are mixed. While the findings of the present study are in accordance with those on a national adult Czech sample [27], they contrast with the results of others showing that thoughts of God did not increase feelings of guilt [34] or the independence of guilt from religious worship [35]. The explanation for this discrepancy seems to be complex. Peterman and LaBelle [19] suggest that higher levels of religious participation in early adolescence could result in a higher sense of personal responsibility and a higher moral sensitivity, which can consequently contribute to feelings of guilt regarding everyday transgressions. This may also be related to a higher perfectionism among religious individuals, as described by Crosby and Bates [36]. However, these researchers also distinguish between adaptive and maladaptive experiencing of both religiosity and perfectionism. Moreover, a higher perfectionism might be a way to deal with scrupulous fears, which have already been associated with anxious attachment to God [37]. Thus, it seems that also the nature of one´s religiosity/spirituality plays a role. Indeed, research has already showed that increased intrinsic religiosity was related to decreased maladaptive perfectionism and consequently to a lower negative affect, while increased extrinsic religiosity increased maladaptive perfectionism [38]. Thus, it seems that a more subtle distinction regarding respondents’ religiosity and spirituality needs to be considered in future research.

In our study, we also found a good internal consistency for both the whole scale as well as for the subscales separately, though the individual items of the scale were mostly only moderately correlated. These findings suggest that the GSES scale represents a short, reliable instrument covering different aspects of the experiences of guilt and shame. This combination makes the tool suitable for implementation into larger surveys, for which classical scenario-based measures are not convenient. Moreover, the scale shows some advantages also when compared to other available statement-based measures, that is, it covers both the guilt and shame aspects and could be used for both the adult and adolescent populations.

### 4.1. Strengths and Limitations

This study has several important strengths, the most important of which is the large representative sample of adolescents with a high response rate and the use of the well-established HBSC methodology. It is also the first validation of the instrument on an adolescent sample and thus offers a new tool for surveys aimed at this age group. A limitation might be that our findings are based on adolescent self-report, which can be inaccurate or influenced by social desirability. Another limitation is a lack of other similar studies, which did not allow for a more detailed comparison. A third limitation is the cross-sectional design of the study, which does not allow us to come to conclusions on causality. A last limitation is a potential decrease of the power of the study regarding sociodemographic differences in religiosity due to a loss of participants.

### 4.2. Implications

Our findings showed a high prevalence of feelings of guilt and shame among adolescents, which suggests that it might be important to address such feelings in a more systematic work with this age group. Thus, improved knowledge in this area would be beneficial, for example, for pedagogists, psychologists, and psychotherapists as well as professional workers in adolescent leisure-time centers. These professionals should be able to understand the differences between guilt and shame and to appropriately distinguish adequate feelings of guilt following a specific misbehavior from a more generalized mixture of guilt and shame that is influencing a whole self-image of an adolescent. They should further implement methods supporting a development of a healthy self-esteem. Moreover, religious educators should be aware that certain forms of religious teaching might be supporting feelings of shame and exaggerated feelings of guilt.

The results of the analyses confirmed the good psychometric characteristics of the GSES on an adolescent representative sample and suggest that it is a suitable tool for measuring these experiences in adolescents. Future research will repeat these analyses on an adult representative sample in order to gain prevalences for mutual comparison. Longitudinal research would be suitable for a better understanding of the developmental aspects of guilt and shame.

## 5. Conclusions

Significantly higher levels of both shame and guilt were observed in girls compared with boys, in students of the ninth grade compared with those of the seventh grade, and in religious adolescents compared with nonreligious ones. Satisfactory results of psychometric analyses show that the GSES is a simple, reliable tool that can be used to analyze feelings of shame and guilt in adolescents.

## Figures and Tables

**Table 1:** Results of a nonparametric comparison of the mean scores of the Guilt and Shame Experience Scale (GSES) and its subscales in groups of adolescents (Wilcoxon rank-sum test and the Kruskal–Wallis test). The *p*-value refers to the comparison of all groups, whereas the relationships given in brackets are the result of multiple group comparisons.

Variables	GSES Mean (SD)	Comparison of GSES between Groups	Shame Mean (SD)	Comparison of Shame between Groups	Guilt Mean (SD)	Comparison of Guilt between Groups
**Gender**						
1. female	2.40 (0.77)	*p* < 0.001	2.25 (0.81)	*p* < 0.001	2.55 (0.84)	*p* < 0.001
2. male	2.00 (0.73)		1.85 (0.74)		2.15 (0.81)	
**Grade**						
1. seventh	2.18 (0.76)	n.s.	2.04 (0.79)	n.s.	2.32 (0.84)	*p* = 0.002
2. ninth	2.21 (0.78)		2.04 (0.81)		2.38 (0.86)	
**Importance of Faith in God**						
1. Not important	2.17 (0.77)	*p* < 0.001	2.02 (0.79)	*p* < 0.001	2.32 (0.85)	*p* < 0.001
2. Important	2.36 (0.78)		2.19 (0.83)		2.52 (0.84)	
**Religious attendance**						
1. Not at all	2.13 (0.77)	*p* < 0.001 (1–2 ***, 1–3 ***, 1–4 ***, 1–5 ***, 1–6 ***, 2–6 **)	1.99 (0.79)	*p* < 0.001 1–2 ***, 1–3 ***, 1–4 **, 1–5 ***, 1–6 ***, 2–6 ***, 5–6 *)	2.28 (0.85)	*p* < 0.001 (1–2 ***, 1–3 ***, 1–4 ***, 1–5 ***, 1–6 ***, 2–6 **)
2. Seldom, rarely	2.33 (0.75)		2.17 (0.78)		2.49 (0.82)	
3. Several times a year	2.40 (0.74)		2.22 (0.78)		2.58 (0.83)	
4. Once a month	2.44 (0.67)		2.24 (0.79)		2.63 (0.73)	
5. Once a week	2.37 (0.75)		2.19 (0.80)		2.54 (0.83)	
6. Several times a week	2.65 (0.88)		2.55 (0.95)		2.76 (0.89)	

Note: SD = Standard Deviation, *** *p* < 0.001, ** *p* < 0.01, * *p* < 0.05, n.s. = Nonsignificant.

**Table 2:** Descriptive characteristics of the three datasets used for the analyses. The two smaller datasets were created from the whole set of data by a random permutation of the respondents’ order and then dividing the permuted dataset into two halves.

Variables		Whole Dataset		1st Half		2nd Half	
				of Permuted Data		of Permuted Data	
		*n*	%	*n*	%	*n*	%
Total		7899		3950		3949	
Gender	female	3895	49.31	1964	49.72	1931	48.90
	male	4004	50.69	1986	50.28	2018	51.10
Grade	seventh	4007	50.73	1985	50.25	2022	51.20
	ninth	3892	49.27	1965	49.75	1927	48.80
Religious attendance	Not at all	5903	74.73	2947	74.61	2956	74.85
	Seldom, rarely	1105	13.99	552	13.98	553	14.00
	Several times a year	323	4.09	164	4.15	159	4.03
	Once a month	112	1.42	62	1.57	50	1.27
	Once a week	273	3.46	136	3.44	137	3.47
	Several times a week	152	1.92	79	2.00	73	1.85
	Missing data	31	0.39	10	0.25	21	0.53
GSES scale raw score	mean (SD)	17.56 (6.19)		17.61 (6.21)		17.51 (6.17)	
	median	17		17		17	
	min–max	8–32		8–32		8–32	
IQR 25%–75%		13–22		13–22		13–21	

Note: SD = Standard Deviation, IQR = Interquartile Range.

**Table 3:** Polychoric correlation coefficients between GSES scale items in the whole dataset.

Item	1	2	3	4	5	6	7	8
1	1.000							
2	0.494	1.000						
3	0.483	0.554	1.000					
4	0.478	0.647	0.579	1.000				
5	0.437	0.317	0.452	0.402	1.000			
6	0.515	0.512	0.545	0.580	0.523	1.000		
7	0.491	0.403	0.550	0.481	0.563	0.608	1.000	
8	0.477	0.491	0.477	0.526	0.405	0.571	0.526	1.000

**Table 4:** Item analysis and factor structure of the GSES scale using exploratory factor analysis.

Item		One-Factor Model		Two-Factor Model			Item Analysis		
		Shame and Guilt	Communality h²	Shame	Guilt	Communality h²	Mean	SD	Correlation with RS without Item
1	I feel guilty, even though I do not know exactly where it is coming from.	0.74	0.55	0.47	0.33	0.55	1.9	0.91	0.64
2	If I do anything wrong, I have to think about it all the time.	0.74	0.55	−0.04	0.93	0.82	2.6	1.02	0.64
3	There are moments when I would rather sink without trace.	0.79	0.63	0.50	0.35	0.62	2.5	1.07	0.69
4	When I do something wrong, I feel an exaggerated feeling of guilt.	0.79	0.63	0.27	0.62	0.68	2.5	1.04	0.70
5	I am losing hope that I will ever be a good person.	0.69	0.48	0.80	−0.07	0.57	1.8	1.02	0.58
6	I blame myself even for things that other people do not think of.	0.84	0.71	0.65	0.24	0.70	2.2	1.07	0.74
7	I experience moments when I cannot even look at myself.	0.80	0.65	0.94	−0.08	0.79	1.9	1.05	0.69
8	I feel the need to explain or apologize for the reasons of my actions.	0.76	0.58	0.49	0.33	0.57	2.2	1.04	0.66
Eigenvalue		4.77		4.77	0.30				
% variability		59.7		59.7	3.7				

Note: SD = Standard Deviation, RS = Raw Score.

**Table 5:** Parameters of the confirmatory factor analysis, the one- and two-factor models of the GSES scale.

		One-Factor Model		Two-Factor Model		
CFA	Item	Loadings	Error Variance	Loading Shame	Loading Guilt	Error Variance
	1	0.714	0.490	0.729	0	0.469
	2	0.778	0.394	0	0.786	0.350
	3	0.786	0.382	0.806	0	0.498
	4	0.826	0.318	0	0.835	0.321
	5	0.696	0.515	0.708	0	0.382
	6	0.829	0.313	0	0.840	0.303
	7	0.807	0.348	0.824	0	0.294
	8	0.736	0.459	0	0.744	0.446
**Model Fit**		**1-Factor Model**		**2-Factor Model**		
Chi-square		554.1 (df 20)		491.6 (df 19)		
*p*-value		< 0.001		< 0.001		
CFI		0.993		0.994		
TLI		0.990		0.990		
RMSEA		0.082		0.079		
SRMR		0.050		0.047		

Note: CFA = Confirmatory Factor Analysis; CFI = Comparative Fit Index; TLI = Tucker–Lewis index; RMSEA = Root Mean Square Error of Approximation; SRMR = Standardized Root Mean Square Residual.

## Supplementary Materials

The following are available online at https://www.mdpi.com/1660-4601/17/23/8901/s1, Supplemenaty File S1—the GSES scale
