# Supplementary material for: A Psychometric Evaluation of the Guilt and Shame Experience Scale (GSES) on a Representative Adolescent Sample: A Low Differentiation between Guilt and Shame
Source: Int J Environ Res Public Health. 2020 Nov 30;17(23):8901. doi: 10.3390/ijerph17238901 (PMC7729781; doi:10.3390/ijerph17238901)
Supplement: Supplementary file 1 [file ijerph-17-08901-s001.pdf]

Guilt and Shame Experience Scale (GSES)

Malinakova, K.; Cerna, A.; Furstova, J.; Cermak, I.; Trnka, R.; Tavel, P. Psychometric Analysis of the Guilt and Shame Experience Scale (GSES). *Cesk. Psychol.* **2019**, *63*, 177–192.

Correspondence:
Klára Maliňáková, Mgr.
OUSHI – Institute of Social Health
Palacký University Olomouc
Univerzitní 244/22, 771 11 Olomouc
Czech Republic
E-mail: klara.malinakova@oushi.upol.cz

To what degree do you agree with the following statement?

		Not at all	A little bit	Somewhat	Significantly
1.	I feel guilty, even though I do not know exactly where it is coming from.	1	2	3	4
2.	If I do anything wrong, I have to think about it all the time	1	2	3	4
3.	There are moments when I would rather sink without trace.	1	2	3	4
4.	When I do something wrong, I feel an exaggerated feeling of guilt.	1	2	3	4
5.	I am losing hope that I will ever be a good person.	1	2	3	4
6.	I blame myself even for things that other people do not think of.	1	2	3	4
7.	I experience moments when I cannot even look at myself.	1	2	3	4
8.	I feel the need to explain or apologize for the reasons of my actions.	1	2	3	4

Subscales:
Items 1, 3, 5 and 7 belong to the Shame subscale.
Items 2, 4, 6 and 8 belong to the Guilt subscale.
